# Psychological distress in women with primary and secondary infertility: a comparative analysis of depression, anxiety, and stress

**DOI:** 10.3389/fpubh.2025.1703256

**Published:** 2025-12-04

**Authors:** Shraddha Chaurasiya, Royana Singh, Bajarang Bahadur, Surbhi Singh, Varsha Maurya, Sangeeta Rai

**Affiliations:** 1 Department of Anatomy, Institute of Medical Sciences, Banaras Hindu University, Varanasi, India; 2 Centre of Biostatistics, Institute of Medical Sciences, Banaras Hindu University, Varanasi, India; 3 Department of Obstetrics and Gynecology, Institute of Medical Sciences, Banaras Hindu University, Varanasi, India

**Keywords:** polycystic ovary syndrome (PCOS), recurrent pregnancy loss (RPL), infertility, psychological distress, mental health, women’s health

## Abstract

**Background:**

Polycystic Ovary Syndrome (PCOS) and Recurrent Pregnancy Loss (RPL) are reproductive disorders frequently linked to psychological distress. This study compared the severity of depression, anxiety, and stress levels between women with PCOS and RPL and assessed their association with years of marriage.

**Materials and methods:**

A cross-sectional study conducted a random sampling method on 157 women (PCOS: 70; RPL: 87) attending the Obstetrics and Gynecology OPD at IMS BHU, Varanasi. Psychological status was assessed using the DASS-21 scale, a reliable tool widely used in reproductive health research. Chi-square test and independent sample t-tests were used for statistical analysis.

**Results:**

Mean scores were significantly higher in RPL than in PCOS: depression (10.7 ± 3.5 vs. 8.8 ± 4.3, p = 0.003), anxiety (13.3 ± 3.8 vs. 10.7 ± 5.5, p = 0.001), and stress (12.3 ± 4.4 vs. 10.3 ± 6.1, p = 0.007). Marriage duration was significantly longer among women with severe depression (RPL: 8.4 ± 5.2 yrs.; PCOS: 6.3 ± 3.1 yrs), anxiety (RPL: 8.6 ± 5.1 yrs.; PCOS: 7.1 ± 3.3 yrs), and stress (RPL: 9.0 ± 5.2 yrs.; PCOS: 6.3 ± 3.1 yrs), all p < 0.05. Age was higher in RPL patients (p = 0.024); LH was higher in PCOS (p = 0.000). No significant differences were observed in AMH or BMI. Psychological symptoms were more severe among women with RPL, and a longer duration of marriage was associated with greater symptom severity. Marriage duration was significantly higher in women classified with severe depression, anxiety, and stress based on DASS-21 scores, indicating a correlational rather than causal relationship.

**Conclusion:**

Routine psychological assessment is recommended for women with PCOS and RPL. Early identification of psychological distress may help improve overall reproductive and emotional health outcomes in these patients.

## Graphical abstract

**Figure:**
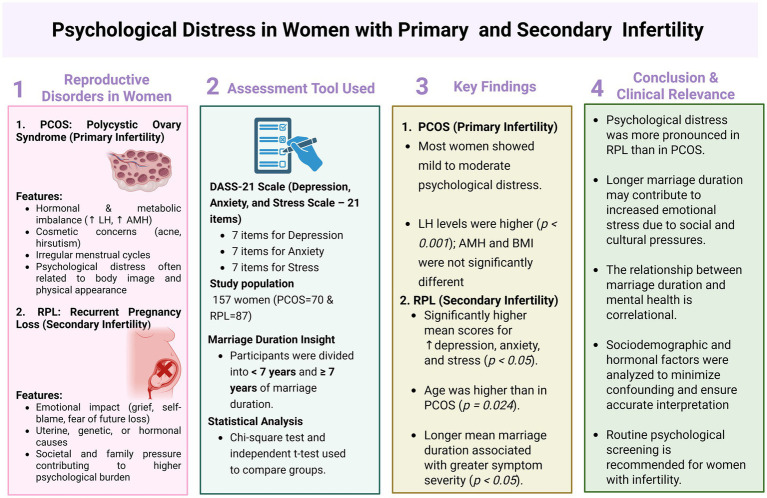

## Introduction

Polycystic Ovary Syndrome (PCOS) and Recurrent Pregnancy Loss (RPL) are common gynecological conditions that substantially affect women’s well-being and reproductive health. PCOS, which impacts 11–13% of reproductive-age women worldwide, and the prevalence of PCOS was close to 10% using Rotterdam’s criteria in India Rotterdam’s criteria, or/and Androgen Excess Society (AES) (1, 2), is characterized by excess androgen production, irregular ovulation, and ovaries with multiple cysts. This endocrine disorder presents various symptoms, such as irregular menstrual cycles, abnormal hair growth, weight gain, and acne. It links metabolic issues, including insulin resistance, obesity, and heightened risks of type 2 diabetes and cardiovascular disease (3, 4). RPL, on the other hand, is defined as the loss of two or more successive pregnancies and affects about 7.46% of women trying to conceive in India and Eleje et al. study shows affects 2–5% globally according to the American Society for Reproductive Medicine (5, 6). RPL can be emotionally traumatic for couples. Both conditions not only present physical challenges but also lead to considerable psychological distress, including symptoms of depression, anxiety, and stress (7).

Jannink et.al shows in his study that the chronic nature of PCOS, coupled with its impact on physical appearance and fertility, can contribute to depression and anxiety (8). The repeated losses associated with RPL can lead to grief, loss, and even post-traumatic stress disorder (PTSD) explained in Yang et al. study (9, 10). The interplay of biological, psychological, and social factors contributes to poor mental health outcomes in women with reproductive disorders. Hormonal imbalances, genetic predispositions, and metabolic disturbances create biological vulnerability, while societal stigma, marital stress, and fertility-related anxiety exacerbate emotional distress (11). Together, these interconnected factors form the biopsychosocial distress observed in PCOS and RPL. Despite growing global research, there remains limited evidence of Indian data exploring psychological distress among women with PCOS and RPL. This study addresses this gap by providing a comparative evaluation of depression, anxiety, and stress within this population using the DASS-21 scale.

### PCOS as infertility: psychological stress

Polycystic Ovary Syndrome (PCOS), a common endocrine disorder, can significantly impact a woman’s fertility and overall wellbeing. While PCOS itself does not always cause infertility, it is a common contributing factor. It is often associated with primary infertility, as many women with PCOS struggle to conceive due to irregular ovulation or anovulation (12). In the study of Parua et al. shows hormonal imbalances and metabolic issues associated with PCOS can disrupt ovulation, making it more challenging to conceive. This struggle with fertility can be a significant source of psychological stress (13). Women with PCOS may experience feelings of frustration, disappointment, and anxiety related to their ability to have children. The visible symptoms of PCOS, such as abnormal hair growth, acne, and weight gain, can also contribute to psychological distress, impacting self-esteem and body image (14, 15). Concerns about long-term health risks related to PCOS, such as diabetes and cardiovascular disease, can further exacerbate anxiety and stress. Wang et al. explained emotional burden of PCOS-related infertility can lead to or worsen existing mental health conditions like depression and anxiety (16, 17).

### RPL as infertility: psychological stress

Recurrent Pregnancy Loss (RPL) is defined as the loss of two or more consecutive pregnancies before 20–24 weeks of gestation, as per the American Society for Reproductive Medicine (ASRM) guidelines (18, 19). Beyond the physical implications, RPL carries substantial mental health concerns, often leading to significant stress. In the Slot et al. study shows the repeated experience of loss can trigger a cascade of negative emotions, including grief, anger, guilt, and a profound sense of loss. Each pregnancy attempt after experiencing RPL can be fraught with anxiety and fear, creating a cycle of hope and disappointment (20). This constant anticipation of loss can lead to chronic stress, impacting both mental and physical well-being. The emotional toll can strain relationships, as partners may grieve differently or struggle to understand the depth of the loss. The psychological burden associated with RPL can manifest in various ways, including difficulty sleeping, changes in appetite, social withdrawal, and even symptoms of depression and anxiety. The cumulative effect of repeated losses can be particularly traumatic, increasing the risk of post-traumatic stress disorder (PTSD) (18, 21).

This study aims to bridge this gap by analyzing the psychological distress associated with PCOS and RPL using the DASS-21 scale. By comparing various demographic, clinical, and psychological parameters, this study seeks to provide a comprehensive understanding of the interplay between these variables.

## Methodology

### Study design and participants

This cross-sectional observational study using random sampling among women with infertility was conducted from August 2023 to August 2024. It included 157 women recruited from the outpatient department (OPD) of the Department of Obstetrics and Gynecology, Institute of Medical Sciences, Banaras Hindu University (IMS BHU). The study involved the collection of primary data directly from the participants. Participants were categorized into two groups: the PCOS Group (*n* = 70) and the Inclusion Criteria for the PCOS group, which were based on the Rotterdam criteria. At the same time, RPL Group (*n* = 87) included women with a history of at least two consecutive pregnancy losses.

### Inclusion and exclusion criteria

- Inclusion criteria

Age range: 18–40 years, PCOS diagnosis was confirmed by a gynecologist using the Rotterdam Criteria, requiring at least two of the following: (i) oligo/anovulation, (ii) clinical or biochemical hyperandrogenism, and (iii) polycystic ovarian morphology on ultrasound. Participants with only polycystic ovaries on ultrasound but without other features were excluded to differentiate PCO from PCOS as shown in Flowchart 1, RPL group history of two or more consecutive pregnancy losses confirmed by medical records as shown in Flowchart 2.

- Exclusion criteria

**FLOWCHART 1:**
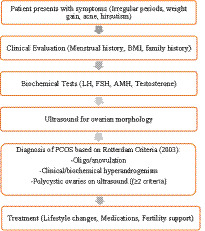
PCOS diagnostic and management pathways.

**FLOWCHART 2:**
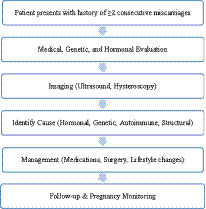
RPL diagnostic and management pathways.

Presence of chronic systemic diseases such as diabetes mellitus, thyroid disorders, or autoimmune conditions, use of hormonal treatments or medications affecting metabolic parameters within the past 3 months, history of uterine anomalies, or other structural causes of infertility.

### Data collection

Data were collected through structured interviews, self-administered questionnaires, and medical record reviews. A standardized general information form was used, which consisted of two sections:

1. *Demographic and Socioeconomic Information*: Age, education level, residence, occupation, family structure, food habits, and duration of marriage.
2. *Infertility-Related Characteristics*: Duration of infertility, Clinical information, including menstrual history (regularity, cycle length), family history of PCOS or pregnancy loss, and Body Mass Index (BMI), Anti-Müllerian Hormone (AMH) to evaluate ovarian reserve, Follicle-Stimulating Hormone (FSH), and Luteinizing Hormone (LH) measured on the second of the menstrual cycle were recorded.

### Psychological assessment

The psychological wellbeing of participants was assessed using the Depression, Anxiety, and Stress Scale (DASS-21) (22). This scale was selected for its robust psychometric validity and reliability across diverse populations. It has been previously applied in reproductive health research, making it suitable for evaluating psychological distress among women with PCOS and RPL in the present study (23, 24). This self-reported questionnaire consists of 21 items divided into three subscales: (1) Depression, related to feelings of sadness, loss of interest, hopelessness, and low self-esteem, indicating the degree of psychological distress associated with infertility, (2) Anxiety, focuses on excessive worry, nervousness, and physiological symptoms such as sweating, trembling, and palpitations, every day in women experiencing reproductive health issues, (3) Stress, measures the individual’s perceived difficulty in coping with daily responsibilities, emotional tension, and frustration, Each item was rated on a 4-point Likert scale ranging from 0 (Not at all) to 3 (Often). The total score for each domain ranged from 0 to 21. Based on the cumulative scores, participants were categorized as follows: Mild (0–7), Moderate (8–14), and Severe (>14). Sociodemographic (age, education, marriage duration) and hormonal parameters (LH, AMH, BMI) were included to minimize potential confounding effects. The association between marriage duration and psychological distress was analyzed as correlational rather than causal, using independent sample t-tests and chi-square tests.

The DASS-21 questionnaire was administered in person in Hindi using a validated translation to ensure better comprehension and cultural appropriateness among participants through face-to-face interviews by research staff in a private area of the outpatient department. Participants were briefed about the purpose of the study, assured of confidentiality, and provided adequate time to complete the responses. To investigate the relationship between duration of marriage and psychological distress, participants were asked to report the total number of years they had been married at the time of assessment. This variable was analyzed for the severity of depression, anxiety, and stress symptoms using the DASS-21 scale. Based on the observed distribution patterns, marriage duration was categorized as less than 7 years, predominantly with mild psychological symptoms, and 7 years or more, associated with moderate to severe symptoms. This threshold was determined by the comparative analysis of mean marital duration across severity levels in both the PCOS and RPL groups. The objective was to ascertain whether longer marriage duration, as a proxy for prolonged exposure to reproductive challenges and sociocultural expectations, was linked to an increased psychological burden.

### Ethics approval

This is a cross-sectional observational study. The Research Ethics Committee of the Institute of Medical Sciences, Banaras Hindu University, approved the study (Approval Code: Dean/2023/EC/6143). Relevant guidelines and regulations are followed in all methods. Informed consent was obtained from all subjects and/or their legal guardians.

### Statistical analysis

Statistical analysis was conducted by using SPSS version 26.0. Participant characteristics were analyzed by descriptive statistics, with continuous variables represented as mean ± standard deviation and categorical variables as frequencies and percentages. Independent *t*-tests were applied to compare mean differences for continuous variables between the PCOS and RPL groups. In contrast, chi-square tests were used to show the associations between categorical variables. Statistical significance was set at *p* < 0.05. This methodological approach ensures the reliability and accuracy of the study findings, providing comprehensive insights into the interrelationship between PCOS, recurrent pregnancy loss, and associated hormonal and psychological factors.

## Results

### Socio-demographic characteristics of participants between PCOS and RPL groups

Table 1 presents the demographic characteristics of participants in the PCOS and RPL groups. There were no significant differences in education levels (*p* = 0.126), residence (*p* = 0.755), occupation (*p* = 0.646), family structure (*p* = 0.121), and food habits (*p* = 0.574) between the two groups. However, a significantly higher proportion of PCOS patients had irregular menstrual cycles (88.6%) compared to the RPL group (20.7%; *p* = 0.000).

**Table 1:** Association of the socio-demographic characteristics with type of infertility patients.

Variable	Category	PCOS (*n* = 70)	RPL (*n* = 87)	Chi-square	*p*-value
Education	Primary education	9 (12.9%)	10 (11.5%)	5.728	0.126
	High school to Intermediate	20 (28.6%)	12 (13.8%)		
	Graduate and above	26 (37.1%)	42 (48.3%)		
	Illiterate	15 (21.4%)	23 (26.4%)		
Residence	Rural	45 (64.3%)	58 (66.7%)	0.097	0.755
	Urban	25 (35.7%)	29 (33.3%)		
Type of period	Irregular	62 (88.6%)	18 (20.7%)	71.522	0.000*
	Regular	8 (11.4%)	69 (79.3%)		
Occupation	Housewife	62 (88.6%)	79 (90.8%)	0.211	0.646
	Private job	8 (11.4%)	8 (9.2%)		
Family history	Blood pressure	16 (22.9%)	13 (14.9%)	25.730	0.000*
	Diabetes	11 (15.7%)	11 (12.6%)		
	Hypertension	18 (25.7%)	9 (10.3%)		
	No	17 (24.3%)	53 (60.9%)		
	Thyroid	8 (11.4%)	1 (1.1%)		
Family structure	Joint	47 (67.1%)	68 (78.2%)	2.403	0.121
	Nuclear	23 (32.9%)	19 (21.8%)		
Food habits	Non-veg	28 (40.0%)	31 (35.6%)	0.315	0.574
	Veg	42 (60.0%)	56 (64.4%)		

*Significance values, *p* < 0.05 considered significant. Bold values indicate statistically significant.

**GRAPH 1:**
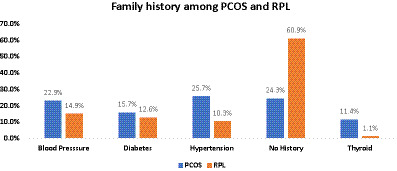
Bar chart titled “Family history among PCOS and RPL” comparing percentages of conditions between PCOS (blue) and RPL (orange):

Family history of hypertension (25.7% in PCOS vs. 10.3% in RPL, p = 0.000), thyroid disorders (11.4% in PCOS vs. 1.1% in RPL, *p* = 0.000), and blood pressure issues (22.9% in PCOS vs. 14.9% in RPL, *p* = 0.000) were more prevalent in the PCOS group. In contrast, a more significant percentage of RPL patients had no family history of medical conditions (60.9%) compared to the PCOS group (24.3%; *p* = 0.000; Graph 1).

### DASS-21 scale assessment of depression, anxiety, and stress between PCOS and RPL groups

In Table 2, women in the RPL group exhibited a significantly higher prevalence of psychological symptoms compared to those with PCOS. In terms of depression, 72.4% of RPL participants were categorized as having moderate symptoms, and 13.8% experienced severe depression, compared to 52.9 and 8.6%, respectively, in the PCOS group. A higher percentage of PCOS women (38.6%) had only mild depression, whereas this was markedly lower in the RPL group (13.8%; *p* = 0.002). Anxiety levels showed a similar trend, with severe anxiety present in 42.5% of RPL participants compared to 28.6% in the PCOS group. Mild anxiety was reported in only 8% of RPL women, as opposed to 32.9% in the PCOS group (*p* = 0.000). Stress levels were also significantly elevated in the RPL group, where 57.5% showed moderate and 33.3% severe stress, in contrast to 32.9% moderate and 34.3% severe stress in the PCOS group. Mild stress was again more common in PCOS (32.9%) than in RPL (9.2%; *p* = 0.000). Based on DASS-21 severity scores, participants with severe levels of depression, anxiety, and stress had a longer mean duration of marriage compared to those with mild or moderate levels. This relationship represents a correlation, not a causal effect.

**Table 2:** Distribution of depression, anxiety, and stress severity levels (DASS-21) in PCOS and RPL groups.

Psychological factor	Category	PCOS (*n* = 70)	RPL (*n* = 87)	Chi-square	*p*-value
Depression	Mild	27 (38.6%)	12 (13.8%)	12.839	0.002*
	Moderate	37 (52.9%)	63 (72.4%)		
	Severe	6 (8.6%)	12 (13.8%)		
Anxiety	Mild	23 (32.9%)	7 (8.0%)	15.603	0.000*
	Moderate	27 (38.6%)	43 (49.4%)		
	Severe	20 (28.6%)	37 (42.5%)		
Stress	Mild	23 (32.9%)	8 (9.2%)	16.064	0.000*
	Moderate	23 (32.9%)	50 (57.5%)		
	Severe	24 (34.3%)	29 (33.3%)		

*Significance values, *p* < 0.05 considered significant. Bold values indicate statistically significant.

### Clinical and hormonal parameters

Table 3 compares the clinical and psychological characteristics of women with PCOS and those with RPL. The mean age was significantly higher in the RPL group (28.6 ± 4.4 years) compared to the PCOS group (27.1 ± 3.4 years, *p* = 0.024). Similarly, years of marriage were significantly longer in RPL women (6.7 ± 4.4 years) than in PCOS (5.2 ± 3.4 years, *p* = 0.022).

**Table 3:** Comparison of different parameter with PCOS and RPL patients.

Variable	PCOS	RPL	*t*-value	*p*-value
Age	27.1 ± 3.4	28.6 ± 4.4	−2.286	0.024*
AMH	7.8 ± 6.2	4.3 ± 3.5	1.203	0.246
BMI	25.2 ± 3.4	24.5 ± 3	1.462	0.146
FSH	6 ± 2.1	6.1 ± 1.9	−0.466	0.642
LH	13.5 ± 6.5	8.8 ± 3.8	5.616	0.000*
Years of marriage	5.2 ± 3.4	6.7 ± 4.4	−2.322	0.022*
Depression Score	8.8 ± 4.3	10.7 ± 3.5	−3.043	0.003*
Anxiety Score	10.7 ± 5.5	13.3 ± 3.8	−3.413	0.001*
Stress Score	10.3 ± 6.1	12.3 ± 4.4	−2.714	0.007*

*Significance values, *p* < 0.05 considered significant. Bold values indicate statistically significant.

Among hormonal markers, LH levels were significantly elevated in the PCOS group (13.5 ± 6.5 mIU/mL) compared to the RPL group (8.8 ± 3.8 mIU/mL, *p* = 0.000). No statistically significant differences were observed in FSH (PCOS: 6.9 ± 2.1; RPL: 6.8 ± 2.1; *p* = 0.842), AMH (PCOS: 4.8 ± 2.3 ng/mL; RPL: 4.7 ± 2.1 ng/mL; *p* = 0.784), or BMI (PCOS: 25.7 ± 4.9 kg/m^2^; RPL: 25.1 ± 4.1 kg/m^2^; *p* = 0.482).

In terms of psychological assessment using DASS-21, the mean depression score was significantly higher in the RPL group (10.7 ± 3.5) vs. the PCOS group (8.8 ± 4.3, *p* = 0.003). Similarly, anxiety scores (RPL: 13.3 ± 3.8; PCOS: 10.7 ± 5.5; *p* = 0.001) and stress scores (RPL: 12.3 ± 4.4; PCOS: 10.3 ± 6.1; *p* = 0.007) were also significantly elevated in RPL participants.

### The relationship between years of marriage and the severity of depression, anxiety, and stress among women with PCOS and RPL was categorized using the DASS-21 scale

In Table 4, depression in both groups, women with severe depressive symptoms, had a longer duration of marriage. In the PCOS group, marriage duration increased from 4.0 ± 2.9 years in mild cases to 7.3 ± 4.1 years in severe cases (*p* = 0.041). In the RPL group, a similar trend was observed, with severe cases averaging 10.3 ± 6.5 years compared to 4.3 ± 3.9 years in mild cases (*p* = 0.002). Anxiety severity was also associated with marriage duration. PCOS women with severe anxiety had been married for 7.1 ± 3.3 years versus 3.7 ± 3.2 years in mild cases (*p* = 0.003). In RPL, severe anxiety corresponded to 8.6 ± 5.1 years compared to 5.7 ± 5.1 years in mild cases (*p* = 0.001), and a similar pattern was seen for stress; in the PCOS group, severe stress cases had a marriage duration of 6.3 ± 3.1 years, compared to 3.8 ± 3.1 years in mild cases (*p* = 0.036). Among RPL patients, the duration rose from 4.6 ± 4 years (mild) to 9 ± 5.2 years (severe; *p* = 0.001).

**Table 4:** Association of psychological distress severity with duration of marriage in PCOS and RPL groups.

Parameter	Group	Severity	Years of marriage (Mean ± SD)	*p*-value
Depression	PCOS	Mild	4.0 ± 2.9	0.041*
		Moderate	5.7 ± 3.4	
		Severe	7.3 ± 4.1	
	RPL	Mild	4.3 ± 3.9	0.002*
		Moderate	6.4 ± 3.7	
		Severe	10.3 ± 6.5	
Anxiety	PCOS	Mild	3.7 ± 3.2	0.003*
		Moderate	5.0 ± 3.1	
		Severe	7.1 ± 3.3	
	RPL	Mild	5.7 ± 5.1	0.001*
		Moderate	5.1 ± 2.9	
		Severe	8.6 ± 5.1	
Stress	PCOS	Mild	3.8 ± 3.1	0.036*
		Moderate	5.4 ± 3.6	
		Severe	6.3 ± 3.1	
	RPL	Mild	4.6 ± 4.0	0.001*
		Moderate	5.6 ± 3.4	
		Severe	9.0 ± 5.2	

*Significance values, *p* < 0.05 considered significant. Bold values indicate statistically significant.

## Discussion

### Findings and interpretation

The current research identified considerable psychological distress in women diagnosed with reproductive disorders, specifically polycystic ovary syndrome (PCOS) and recurrent pregnancy loss (RPL). According to the DASS-21 evaluation, women experiencing RPL had significantly higher depression, anxiety, and stress scores than those with PCOS, suggesting that repeated pregnancy loss may impose a more profound and acute psychological impact than the chronic reproductive and metabolic challenges associated with PCOS. Similar findings have been reported in recent studies (Wang et al. and Ziedenberg et al.), where RPL was associated with heightened emotional trauma, anticipatory anxiety, and fear of future conception failure compared with women with other infertility etiologies. Longer marital duration was linked to increased levels of depression, anxiety, and stress, with statistical significance particularly evident in the RPL group. This implies that as reproductive difficulties persist unresolved over time, the psychological burden grows (25–27). The observed correlation between longer marriage duration and increased emotional distress may reflect cumulative social and cultural pressures experienced by women facing infertility in Indian society. In the study by Lakatos et al. prolonged marital duration often coincides with repeated treatment failures, family expectations, and societal stigma, which can intensify psychological symptoms. This pattern may reflect the cumulative emotional strain that develops as reproductive challenges remain unresolved. In the Indian sociocultural context, prolonged infertility or repeated pregnancy losses often expose women to sustained familial pressure, social comparison, and stigmatization, all of which can amplify distress (28, 29). Repeated medical treatment and societal expectations surrounding motherhood likely compounded feelings of inadequacy and hopelessness, contributing to higher depression and anxiety scores over the years.

**Figure 1:**
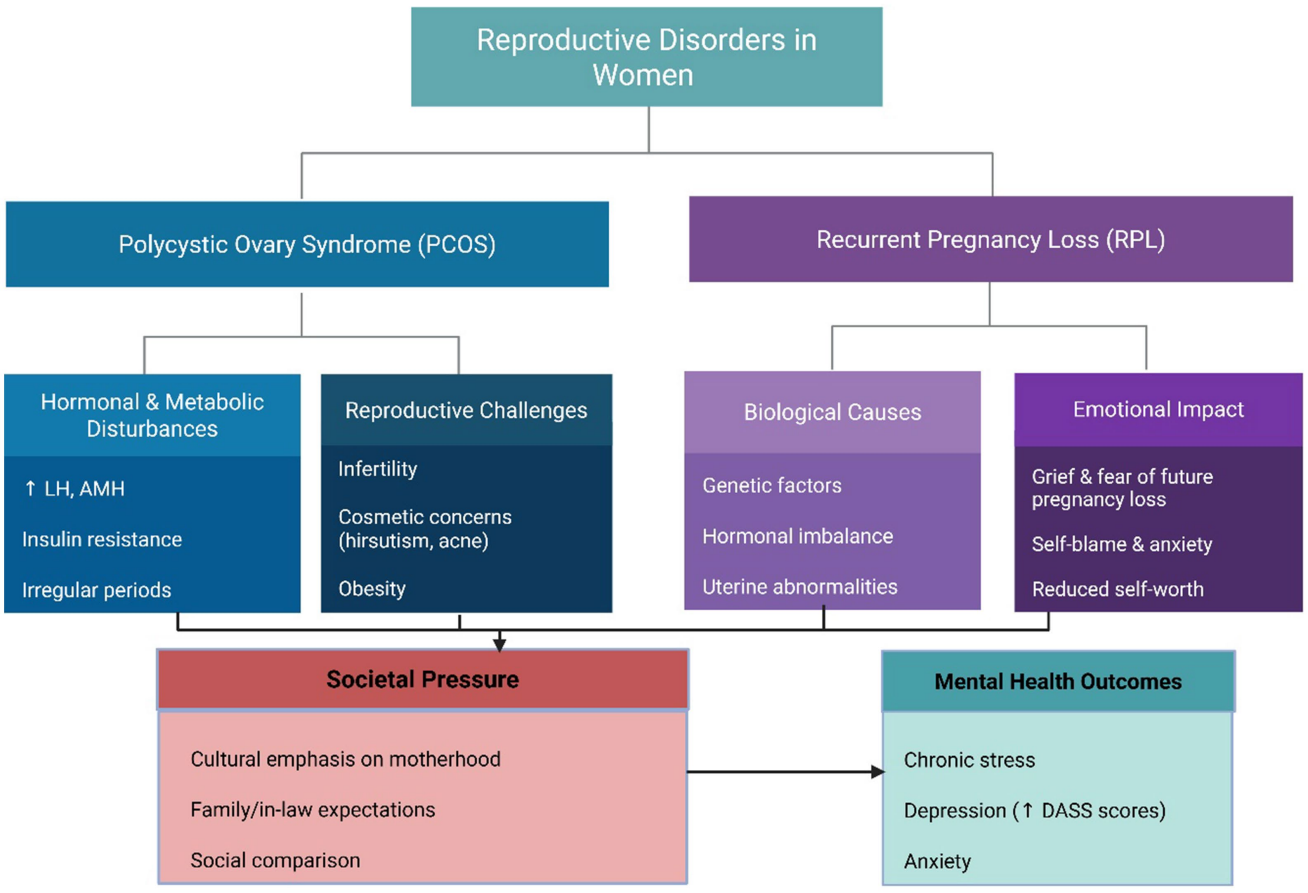
Flowchart depicting reproductive disorders in women, focusing on Polycystic Ovary Syndrome (PCOS) and Recurrent Pregnancy Loss (RPL). PCOS includes hormonal disturbances, reproductive challenges, and societal pressure. RPL covers biological causes and emotional impact. Both disorders lead to mental health outcomes like chronic stress and anxiety.

Furthermore, the observed higher prevalence of metabolic comorbidities, such as diabetes, hypertension, and thyroid disorders, among PCOS participants is consistent with existing literature emphasizing the endocrine-psychological link in PCOS pathophysiology. These comorbidities may contribute to fatigue, body image dissatisfaction, and emotional dysregulation (30). Family history analysis revealed a higher occurrence of diabetes, hypertension, and thyroid disorders among PCOS participants, highlighting the metabolic burden already associated with the condition (31). These findings highlight a recent study by Yu et al. where biopsychosocial interaction wherein prolonged marital duration and persistent reproductive challenges lead to chronic stress, which can dysregulate the hypothalamic–pituitary-ovarian axis. This hormonal imbalance may exacerbate reproductive dysfunction, illustrating how psychological distress and biological factors collectively influence outcomes in PCOS and RPL (32). In the study by Sharma & Shrivastava where clinically, prolonged infertility is often accompanied by a gradual decline in ovarian reserve and oocyte quality, reducing the likelihood of conception and further intensifying psychological distress. These findings are consistent with previous research indicating that both biological and psychosocial factors contribute to heightened emotional burden in women with longer durations of infertility (33). These findings were interpreted in the context of relevant sociodemographic and hormonal factors to ensure a balanced and accurate understanding of the results and the robustness of the findings. The conceptual model shown in Figure 1 visually integrates the biological, emotional, and societal factors contributing to mental health outcomes in women with PCOS and RPL.

### Alignment with prior studies

Our results align with those of earlier studies that showed increased levels of depression, anxiety, and stress in women with reproductive disorders. In the study by Mevorach-Zussman et al. where women with PCOS and recurrent pregnancy loss (RPL) have greater psychological vulnerability (34). However, our findings indicate that women experiencing RPL endured significantly more intense emotional distress than those with PCOS. This could be due to the cumulative trauma of repeated pregnancy loss, which leads to a more profound sense of grief, fear of future failures, and ongoing uncertainty regarding reproductive success.

This study contributes to the existing knowledge by considering emotional outcomes and the influence of marriage duration, a relatively unexplored factor in this area (35). Unlike previous research, which primarily focused on depression or anxiety separately, we assessed all three components of the DASS-21 scale. We discovered a clear pattern: a longer marital duration was linked to more severe psychological symptoms. This is consistent with literature suggesting that extended periods of infertility or repeated pregnancy failures can exacerbate emotional strain over time, particularly in sociocultural contexts where motherhood is closely associated with a woman’s social identity.

Additionally, our findings support the sociocultural view that emotional outcomes in infertility are not solely biological but are influenced by external pressures. In countries such as India, In the study by Miller et al. and Sharma et al. where, societal and familial expectations regarding reproduction are intense, the inability to conceive or maintain a pregnancy often leads to social isolation, blame, or marital tension. Studies have documented these cultural stressors (36, 37). By correlating DASS scores with years of marriage, our study highlights how mental health concerns in PCOS and RPL are intensified by both time and context, emphasizing the urgent need for integrated psychosocial support in reproductive healthcare.

### Clinical implications

The implications of these findings are significant in clinical practice. First, they highlighted the importance of routinely conducting psychological assessments for women experiencing reproductive issues, particularly those with persistent infertility or recurrent pregnancy losses. Instruments such as the DASS-21 (38) can be seamlessly incorporated into healthcare environments to detect women who may be at risk of depression, anxiety, and stress, enabling prompt referrals to mental health professionals (38). Secondly, it is crucial for healthcare providers, especially gynecologists, fertility experts, and endocrinologists, to be trained in identifying signs of emotional exhaustion and marital strain, and to handle consultations with empathy. The psychological aspects of infertility and reproductive loss are often overlooked in medical care, despite their potential impact on treatment compliance, marital relationships, and overall quality of life (39, 40).

Additionally, Aji et al. and Nakao et al. study, where mental health treatments such as cognitive-behavioral therapy (CBT), support groups, and family counseling can assist patients in developing effective coping mechanisms. In societies where a woman’s identity and social standing are closely linked to motherhood, these psychological interventions can significantly alleviate emotional distress and enhance not only mental health outcomes, but also improve the success of assisted reproductive technologies (41, 42).

### Research implications

The findings of the present study underscore the need for longitudinal and interventional research to better understand how psychological distress evolves among women with PCOS and RPL. In the study by Anan et al., the observed relationship between the duration of marriage and emotional burden highlights the necessity to investigate the long-term effects of prolonged infertility-related stress on mental health and treatment outcomes (43, 44).

Furthermore, it is essential to develop and validate culturally sensitive screening tools and intervention frameworks tailored to the Indian sociocultural context. Such tools would enable early identification and management of mental health concerns (45), particularly among women facing extended infertility or repeated pregnancy loss. Integrating standardized psychological assessments into routine gynecological and fertility care protocols may improve emotional well-being and enhance therapeutic compliance and reproductive success rates (45).

Future interventional and longitudinal studies are warranted to examine causal pathways and assess whether targeted psychosocial interventions can reduce distress levels among infertile women over time. Should also adopt qualitative and mixed-method approaches to explore the intricate psychosocial dynamics, coping strategies, and stigma faced by women with reproductive disorders. These insights could guide the development of multidimensional, patient-centered mental health interventions, fostering a more holistic approach to reproductive healthcare (46). Additionally, investigating the role of social support systems, including family and community networks, in mitigating psychological distress could provide valuable insights for developing targeted interventions (47). Exploring the potential benefits of integrating alternative therapies, such as mindfulness-based stress reduction techniques or support group interventions, alongside conventional medical treatments could offer a more comprehensive approach to addressing the emotional needs of women with PCOS and RPL.

### Strengths and limitations

The comparative design is a significant advantage, as it sheds light on the psychological differences between two separate reproductive disorders. The study’s value is enhanced by employing a validated instrument (DASS-21) and considering marriage duration as a psychosocial element (39). Nonetheless, the study’s cross-sectional design limits the ability to draw causal conclusions, and there may be reporting bias due to self-administered questionnaires. Additionally, the study did not include a healthy control group, which limits direct comparison with the general population. However, as this study was designed to compare psychological distress between women with primary (PCOS) and secondary (RPL) infertility, the relative differences within infertility conditions were the primary focus. Future research, including control participants, will help establish broader population-level inferences (40).

## Conclusion

This study demonstrates that psychological distress, particularly depression, anxiety, and stress, is significantly higher in women with recurrent pregnancy loss (RPL) than in those with polycystic ovary syndrome (PCOS). A key finding is the association between longer marriage duration and greater emotional burden, suggesting that persistent reproductive challenges and sociocultural pressures intensify psychological distress. The study contributes uniquely by quantitatively linking marriage duration with DASS-21 scores, emphasizing the need for psychosocial evaluation alongside clinical management. Integrating routine mental health screening and evidence-based interventions such as CBT, IPT, and grief counseling into reproductive care is essential. Healthcare professionals should be trained to recognize emotional distress and provide empathetic, patient-centered support. Future research should adopt longitudinal designs and develop culturally tailored intervention models to understand better and mitigate the psychosocial impact of reproductive disorders like PCOS and RPL, thereby improving women’s overall quality of life.

## Data Availability

The raw data supporting the conclusions of this article will be made available by the authors, without undue reservation.
